# Clinical Diagnosis and Magnetic Resonance Imaging in Patients With Transient and Minor Neurological Symptoms: A Prospective Cohort Study

**DOI:** 10.1161/STROKEAHA.122.039082

**Published:** 2022-08-09

**Authors:** William N. Whiteley, Allan MacRaild, Ying Wang, Martin Dennis, Rustam Al-Shahi Salman, Alasdair Gray, Matthew J. Reed, Catriona Graham, Joanna M. Wardlaw

**Affiliations:** Centre for Clinical Brain Sciences, University of Edinburgh, United Kingdom (W.N.W., A.M., M.D., R.A-.S.S., J.M.W.).; Nuffield Department of Population Health, University of Oxford, United Kingdom (W.N.W.).; Usher Institute, University of Edinburgh, United Kingdom (W.N.W., R.A-.S.S., A.G., M.J.R.).; Emergency Medicine Research Group Edinburgh (EMERGE), Royal Infirmary of Edinburgh, United Kingdom (A.M., A.G., M.J.R.).; Neurology Department in the Second Affiliated Hospital of Kunming Medical University, China (Y.W.).; Edinburgh Clinical Research Facility (C.G.), University of Edinburgh, United Kingdom.; Edinburgh Imaging (J.M.W.), University of Edinburgh, United Kingdom.

**Keywords:** diagnosis, differential, ischemia, magnetic resonance imaging, transient ischemic attack, tomography

## Abstract

The utility of magnetic resonance imaging (MRI) brain in patients with transient or minor neurological symptoms is uncertain. We sought to determine the proportion of participants with transient or minor neurological symptoms who had MRI evidence of acute ischemia at different clinical probabilities of transient ischemic attack (TIA) or minor stroke.

**Methods:**

Cohort of participants with transient or minor neurological symptoms from emergency and outpatient settings. Clinicians at different levels of training gave each participant a diagnostic probability (probable when TIA/stroke was the most likely differential diagnosis; possible when TIA/stroke was not the most likely differential diagnosis; or uncertain when diagnostic probability could not be given) before 1.5 or 3T brain MRI ≤5 days from onset. Post hoc, each clinical syndrome was defined blind to MRI findings as National Institute of Neurological Disorders and Stroke criteria TIA/stroke; International Headache Society criteria migraine aura; non-TIA focal symptoms; or nonfocal symptoms. MRI evidence of acute ischemia was defined by 2 reads of MRI. Stroke was ascertained for at least 90 days and up to 18 months after recruitment.

**Results:**

Two hundred seventy-two participated (47% female, mean age 60, SD 14), 58% with MRI ≤2 days of onset. Most (92%) reported focal symptoms. MR evidence of acute ischemia was found, for stroke/TIA clinical probabilities of probable 23 out of 75 (31% [95% CI, 21%–42%]); possible 26 out of 151 (17% [12%–24%]); and uncertain 9 out of 43, (20% [10%–36%]). MRI evidence of acute ischemia was found in National Institute of Neurological Disorders and Stroke criteria TIA/stroke 40 out of 95 (42% [32%–53%]); migraine aura 4 out of 38 (11% [3%–25%]); non-TIA focal symptoms 16 out of 99 (16% [10%–25%]); and no focal features 1 out of 29 (3% [0%–18%]). After MRI, a further 14 (5% [95% CI, 3–8]) would be treated with an antiplatelet drug compared with treatment plan before MRI. By 18 months, a new ischemic stroke occurred in 9 out of 61 (18%) patients with MRI evidence of acute ischemia and 2 out of 211 (1%) without (age-adjusted hazard ratio, 13 [95% CI, 3–62]; *P*<0.0001).

**Conclusions:**

MRI evidence of acute brain ischemia was found in about 1 in 6 transient or minor neurological symptoms patients with a nonstroke/TIA initial diagnosis or uncertain diagnosis. Methods to determine the clinical and cost-effectiveness of MRI are needed in this population.

Neurologists and stroke physicians find it difficult to rule in or out a diagnosis of minor stroke or transient ischemic attack (TIA) in patients with transient or minor neurological symptoms. In these patients, the best initial investigation strategy is still uncertain.

One strategy is to use magnetic resonance imaging (MRI) of the head for every patient with transient or minor neurological symptoms. MRI of the brain may detect diffusion-weighted imaging (DWI) lesions that are typical of acute brain ischemia or (rarely) may make a positive diagnosis of an alternative cause for symptoms such as multiple sclerosis or brain tumor. Where the diagnosis of TIA or stroke is secure, the pattern of ischemia seen on MRI may help to identify the underlying cause, such as multivascular-territory ischemia in cardioembolism. However, an MRI brain without signs of acute ischemia is the commonest finding in patients with a clinical diagnosis of TIA or minor stroke and may be falsely reassuring.^1,2^ This is particularly likely in patients with brain stem TIA or minor stroke, or with delayed presentation, where normal MRI brain imaging is found more frequently.^2^

To measure the benefits of an MRI strategy that scans all patients rather than a clinically targeted strategy, we need an estimate of the proportion of patients with positive DWI at different levels of clinical probability of a diagnosis of a TIA or minor stroke. For example, if the great majority of patients with a high clinical probability of TIA or stroke had DWI changes, or the great majority of patients with a low clinical probability had a negative MRI, then MRI could be targeted at those with intermediate clinical suspicion. However, if clinical probability did not identify a very high or low probability of DWI lesions, then a policy to scan all patients might be preferred.

We, therefore, sought to recruit a cohort of participants from clinical practice with transient or minor symptoms where TIA or stroke was suspected but not confirmed and to determine the proportion of patients with MRI evidence of acute ischemia at different clinically predicted risks of TIA or minor stroke.

## Methods

Data from this study are available on request from the corresponding author.

We invited adults ≥18 years to participate within 5 days of transient or minor neurological symptoms where TIA or minor stroke was suspected by an assessing clinician. Participants in a major acute teaching hospital were referred to the study team by stroke physicians in the emergency department, acute medical wards, and a TIA clinic (which dealt with emergency department and general practitioner referrals). Symptoms were minor when participants had a National Institutes of Health (NIH) Stroke Scale ≤5 at assessment and were expected to be discharged or spend no more than one night in hospital. Predefined, relevant symptoms included diplopia, vertigo, cognitive complaints of sudden onset, isolated speech disturbance (dysphasia or dysarthria), transient or mild weakness, heaviness or clumsiness of a limb, isolated limb sensory disturbance, hemivisual field disturbance, migraine aura with no prior history of migraine aura ≥50 years of age, or a combination of these symptoms. We excluded potential participants: with a definite clinical diagnosis of TIA or stroke (ie, where the assessing clinician diagnosed TIA or stroke with no differential diagnosis); with monocular symptoms; who had been considered for thrombolysis or thrombectomy; with a contraindication to MRI scanning; who were pregnant; who were not resident in Lothian‚ United Kingdom; or who could not be easily followed-up (eg, no address or telephone number). At presentation, a study nurse collected age, sex, baseline medical and demographic variables, and patients answered a prespecified questionnaire about their symptoms.

Each assessing clinician gave their clinical diagnosis before imaging as probable or possible TIA or stroke, or an uncertain diagnosis. Clinicians made a probable diagnosis where stroke or TIA was the most likely of several differential diagnoses; a possible diagnosis where a noncerebrovascular diagnosis was the most likely and stroke or TIA less likely; and an uncertain diagnosis where a diagnostic probability could not be assigned on a proforma with diagnostic reminder. Each clinician gave their alternative noncerebrovascular diagnoses and planned treatment if MRI imaging was not available.

Post hoc, with review of all preimaging clinical records and blind to MRI results, participants’ symptoms were classified by a stroke neurologist into a TIA or stroke with National Institute of Neurological Disorders and Stroke criteria^3^; a typical migraine aura with or without headache with International Headache Society Criteria^4^; an attack with focal features (ie, could be attributed to dysfunction in one brain area) not in NIH or International Headache Society Criteria criteria; or an attack with no focal features. An ABCD^2^ score (age [A]‚ blood pressure [B]‚ clinical features [C]‚ duration [D] and diabetes [D]) was calculated for each participant.^5^

Each participant had an MRI of the brain within 5 days of symptom onset with 3T or 1.5T scanner with T1, T2, fluid-attenuated inversion recovery (FLAIR)‚ blood sensitive, and DWI sequences. Each MRI was reported by an unmasked clinical radiologist and separately by a masked research radiologist using a standardized proforma. The presence of acute ischemia was defined primarily by the presence of a DWI lesion but also from the appearance of T2 and FLAIR sequences. We also recorded deep and subcortical white matter hyperintensities using the Fazekas scale,^6^ the number of old lacunes, microbleeds, old cortical, large subcortical, and likely small subcortical or posterior fossa infarcts, old hemorrhages, cortical siderosis, superficial, and deep brain atrophy. Other structural lesions likely to have caused the symptoms were also recorded (tumors, subdural hematoma, etc). Where there was disagreement between the clinical and research radiologist, a third radiologist reviewed the imaging and came to a final decision.

At 90 days postevent, we contacted each participant by email or letter depending on preference, and by telephone for those who did not respond. We asked each participant whether they had had further symptoms or a stroke or myocardial infarction and measured participants’ modified Rankin Scale‚ and EuroQol 5-level score. We further assessed the occurrence of further stroke, myocardial infarction, or death by reading each participant’s hospital-based electronic health records up to May 19, 2020 (ie, when the last participant had 90 days of follow-up).

We analyzed data with SAS (V 9.4 SAS Institute, Inc, Cary, NC). We compared the characteristics of groups of participants with and without MRI evidence of acute ischemia, using χ^2^, Fisher exact, or *t* tests as appropriate. We calculated 95% CIs of proportions. To compare the incidence of stroke during follow-up in participants with and without MRI evidence of acute ischemia, we calculated a hazard ratio adjusting for age (with few events, we included only one covariate in this model).^7^ We calculated an area under the receiver operator characteristic curve for a logistic regression model predicting MRI evidence of acute ischemia, using the predictors defined in a previous publication^8^: age, sex, motor or speech symptoms, ongoing symptoms, abnormal neurological examination, and no prior identical symptomatic event. The report is consistent with the Strengthening the Reporting of Observational Studies in Epidemiology guidelines (Table S1, Figure S1).^9^

All participants gave written consent. The East of England (Essex) Research Ethics Committee gave approval for the study (18/EE/0157).

## Results

Between August 18, 2018 and February 19, 2020, 286 people consented to take part; 14 were unable to have an MRI scan (4 claustrophobia, 1 contraindication, 3 too large for scanner, and 6 for >1 reason).

Of the 272 participants, most had an MRI brain ≤2 days of symptom onset (157, 58%). Participants were mostly male (145, 53%); had a mean age of 60 (SD, 14); had only one episode of neurological symptoms (205, 75%); and had mild or no neurological signs (NIH Stroke Scale, 0: 247, 91%), which had either not resolved at presentation (118, 43%) or had resolved but lasted for ≥1 hour (94, 35%). In most participants, symptoms were monosymptomatic or all began simultaneously (156, 57%). Common nonfocal symptoms were tiredness or fatigue (54%), headache (43%), and nausea or sickness (40%; Tables 1 and 2).

**Table 1.**
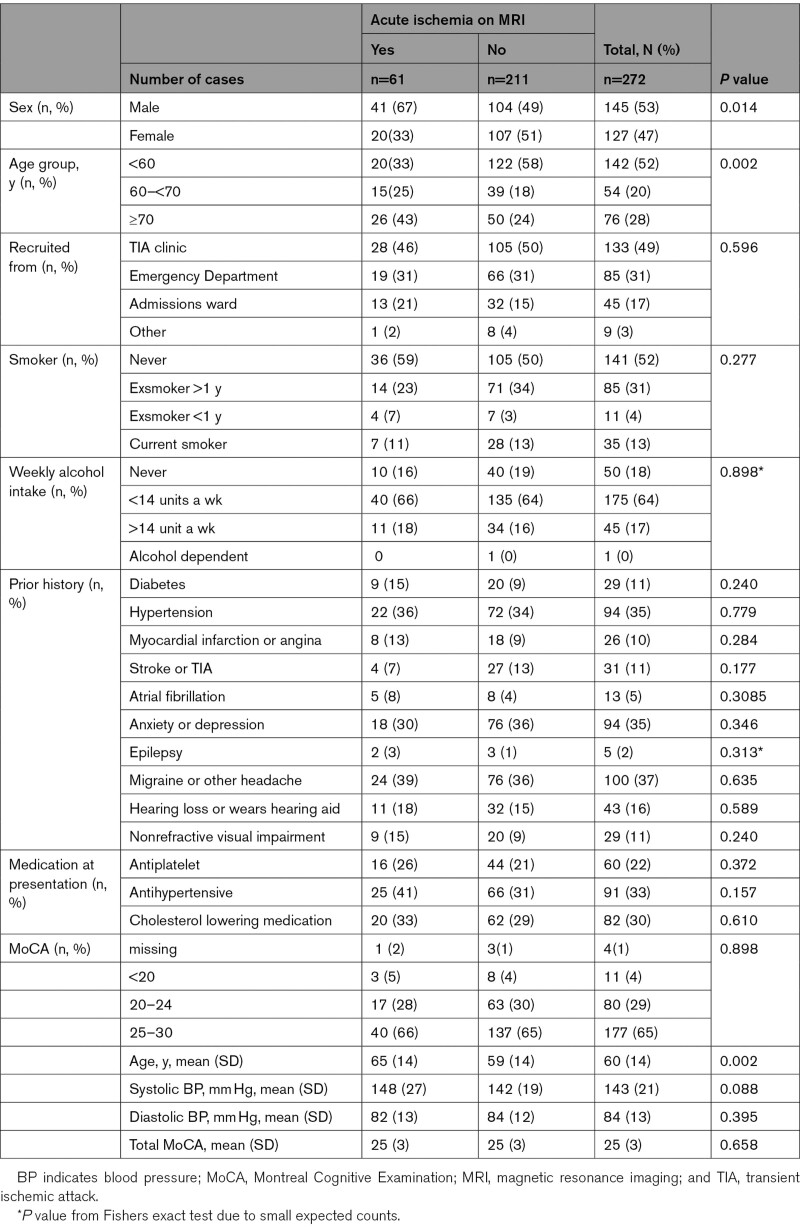
Characteristics of Participants at Recruitment by Presence or Absence of Acute Ischemia on MRI Head

**Table 2.**
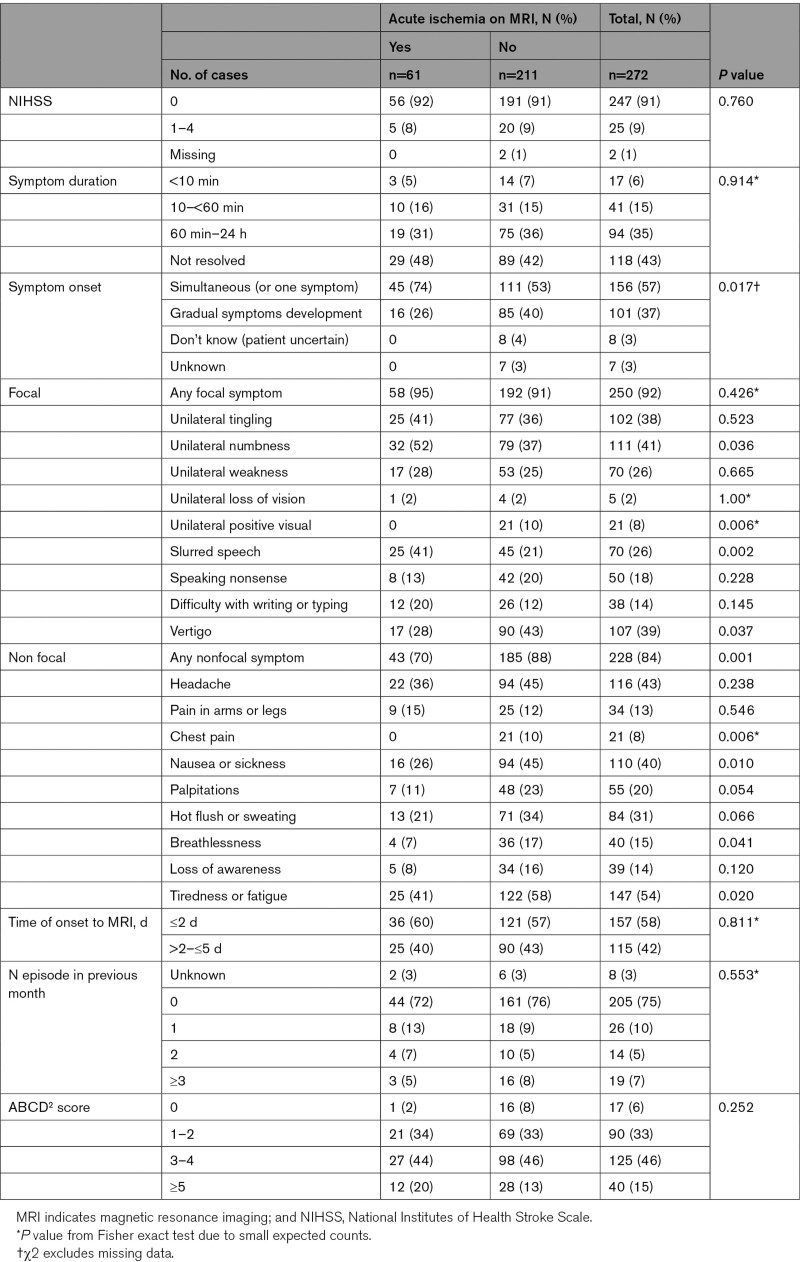
Characteristics of Symptom Onset at Recruitment by Presence or Absence of Acute Ischemia on MRI Head

MRI evidence of acute brain ischemia was present in 61 (22%) participants. Of these 61 participants, 16 (26%) had one cortical infarct, 16 (26%) had one subcortical lacunar infarct, 11 (18%) had one cerebellar or brain stem infarct, and 18 (30%) had >1 infarct. The acute infarcts were all thought to be relevant to the participant’s symptoms.

The absolute differences in clinical characteristics between participants with and without MR evidence of acute ischemia were modest: 33% versus 51% female (*P*=0.0135) and mean age 65 years versus 59 years (*P*=0.002), respectively. Focal symptoms were frequent in both groups (95% versus 91%, *P*=0.4264), although some symptoms were more frequent in participants with MRI evidence of acute brain ischemia: simultaneous onset of more than one symptom or one symptom type (74% versus 53%, *P*=0.017); unilateral numbness (52% versus 37%, *P*=0.036); and slurred speech (41% versus 21%, *P*=0.002). Vertigo was less frequent in participants with an acute infarct on imaging (28% versus 43%, *P*=0.037). Nonfocal symptoms were frequent in participants with and without MRI evidence of acute ischemia (70% versus 88%, *P*=0.001), but individual nonfocal symptoms were less frequent in participants with MRI evidence of acute ischemia: nausea or sickness (26% versus 45%, *P*=0.010), breathlessness (7% versus 17%, *P*=0.041), and tiredness or fatigue (41% versus 58%, *P*=0.020; Tables 1 and 2).

Participants with an acute infarct on imaging had more evidence of cerebral small vessel disease and brain atrophy. They had more white matter hyperintensities that were deep (*P*=0.0307) or periventricular (*P*=0.0104); atrophy that was deep (*P*=0.0015) or superficial (*P*=0.0199); old infarction (52% versus 25%, *P*<0.0001) and more lacunes (Table 3).

**Table 3.**
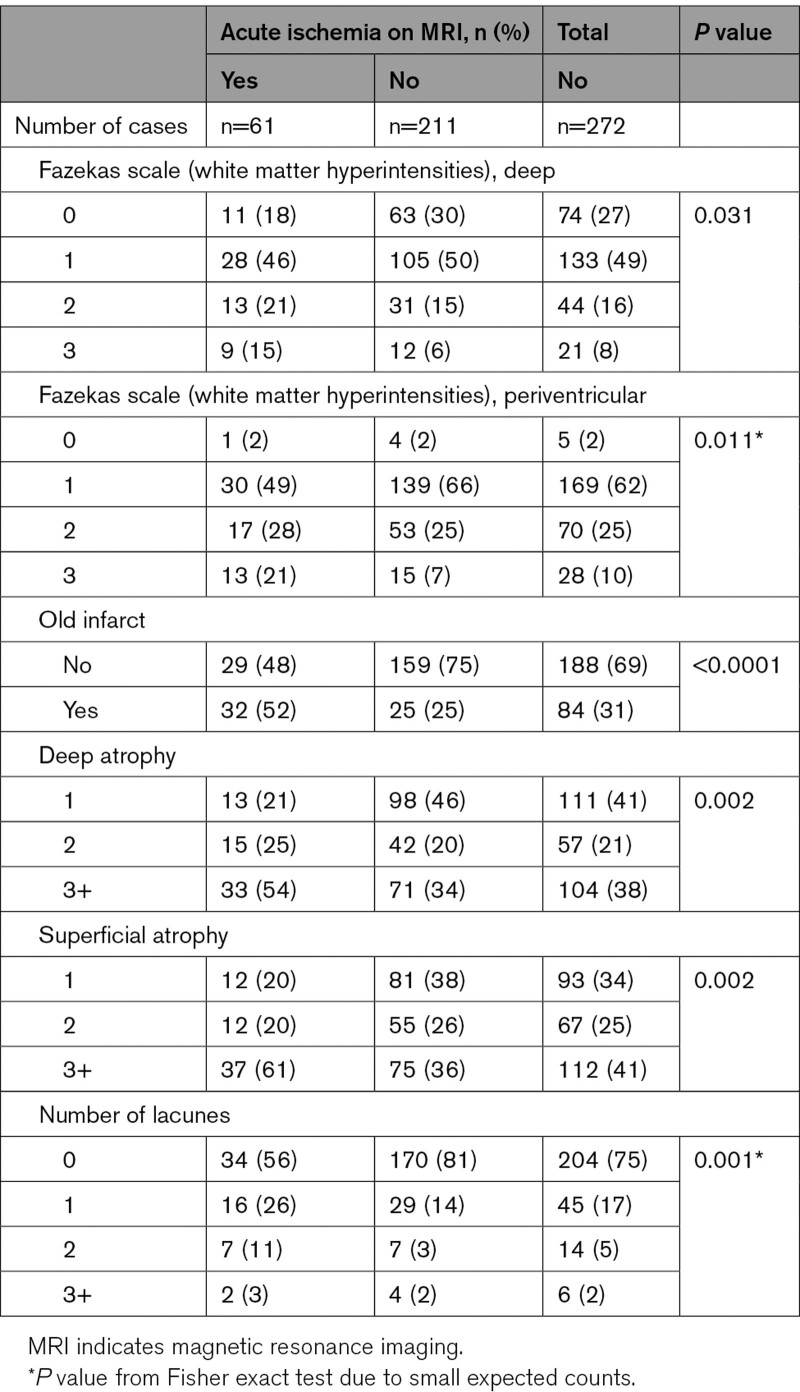
Other MRI Findings by Presence of MRI Detected Acute Ischemia

Assessing clinicians gave their clinical suspicion of stroke or TIA before imaging. These clinicians were: consultants 104, 38%, trainees 166, 61% whose primary speciality was stroke medicine 158, 58%, geriatrics 43, 16%, and neurology 62, 23%. Before brain imaging, diagnoses were uncertain (16%); definite TIA or stroke (1%); probable TIA or stroke (28%); or possible TIA or stroke (55%). The differential diagnoses were migraine (23%); unknown (21%); vertigo (15%); functional neurological disorder (10%); neuropathy (5%); seizure (4%); anxiety (2%); and other diagnoses (21%).

**Figure 1.**
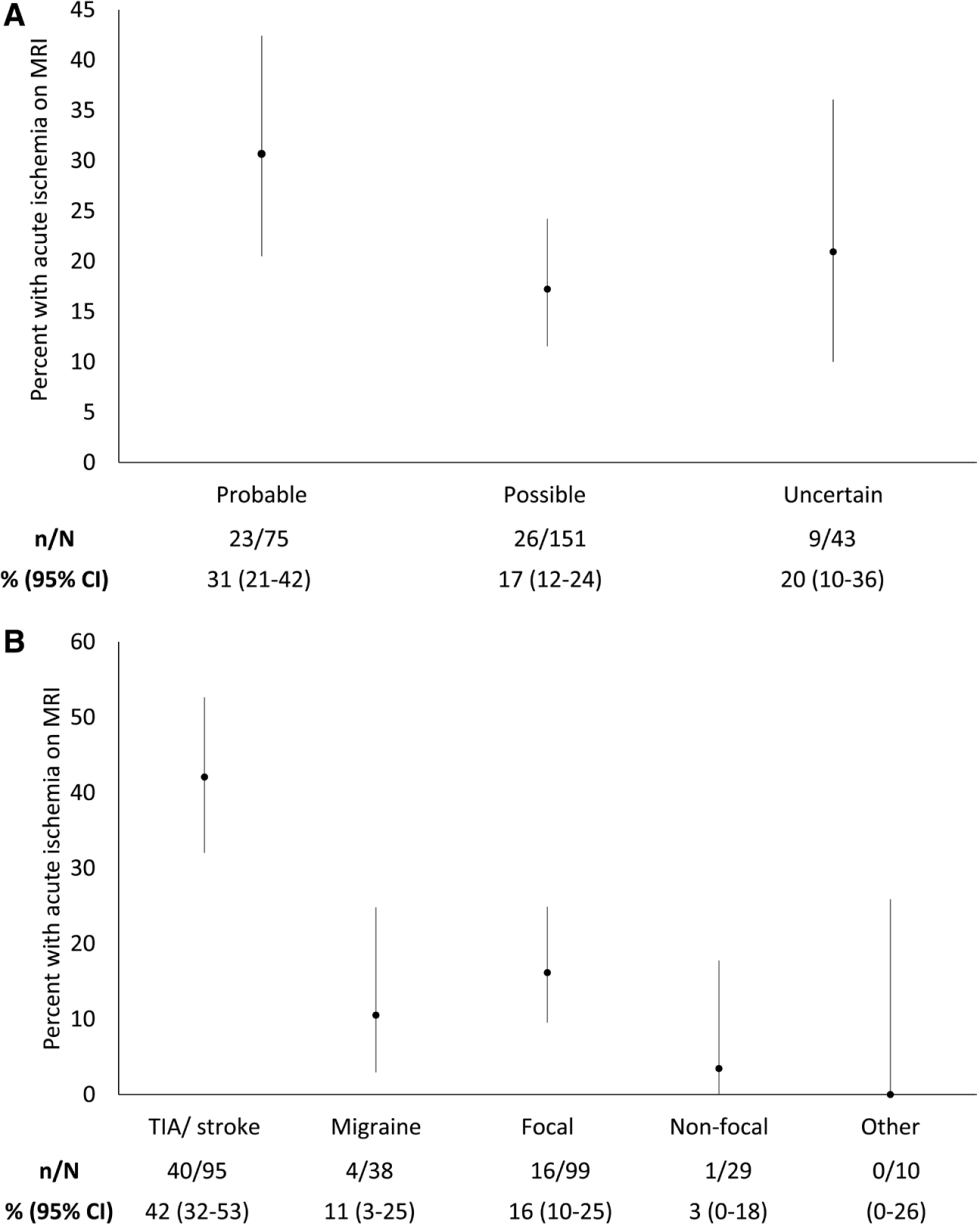
**Percentage of participants with magnetic resonance imaging (MRI) evidence of acute ischemia. A**, With a prospectively provided clinical diagnosis of probable, possible, or uncertain stroke or transient ischemic attack (TIA; χ^2^ *P*=0.07). **B**, With a retrospective diagnosis of TIA or stroke by National Institute of Neurological Disorders and Stroke (NINDS) definition; migraine; focal non-NINDS attacks; nonfocal symptoms; or other diagnosis.

The proportion of participants with MRI evidence of acute ischemia in each diagnostic group was for: probable stroke or TIA, 31% (95% CI, 21%–42%); possible stroke or TIA 17%, (95% CI, 12%–24%); and uncertain, 20% (95% CI, 10%–36%; χ^2^ *P*=0.07; Figure 1A) Three participants were recruited in error with a definite diagnosis of TIA or stroke, all of whom had MR evidence of acute ischemia.

Post hoc, the diagnosis of symptoms after review of records blind to imaging were: TIA/stroke by NIH criteria (95, 35%) non-NIH focal symptoms (99, 36%); migraine by International Headache Society Criteria criteria (38, 14%); no focal features (29, 11%); and other diagnosis (10, 4%). The proportion of participants with MR evidence of acute ischemia in each group was: NIH criteria TIA or stroke, 42% (32%–53%); migraine by International Headache Society Criteria criteria, 11% (3%–25%); NIH focal symptoms, 16% (10%–25%); and nonfocal features, 3% (0%–18%) and other diagnosis 0% (Figure 1B) The distribution of the ABCD^2^ score was similar in participants with and without MRI evidence of acute ischemia (*P*=0.252). A logistic regression model with covariates (age, sex, motor/speech symptoms, ongoing symptoms, abnormal initial neurological exam, prior identical symptomatic event) from a previous study^8^ had only modest discrimination (C statistic, 0.70 [95% CI, 0.62–0.77]).

Before assessment, 60 (22%) of participants were taking an antiplatelet. Before clinicians had access to MRI scanning, they said they would treat a further 142 (52%) with an antiplatelet for at least a month, whatever the MRI results. If all participants with MRI evidence of acute ischemia would be treated with an antiplatelet, a further 14 (5% [95% CI, 3–8]) would be treated with an antiplatelet after MRI.

We followed 264 (97%) participants for up to 90 days by email, post, or telephone. By 90 days, 4 participants, all with MRI evidence of acute ischemia at baseline, had a subsequent ischemic stroke.

**Figure 2.**
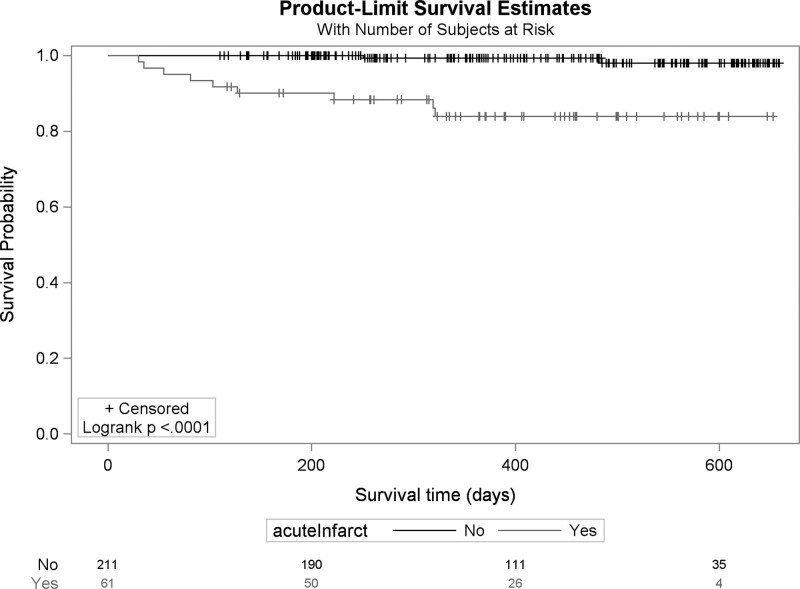
Kaplan-Meier survival plot for the incidence of stroke during follow-up after magnetic resonance (MR) brain images, in participants with and without MR evidence of acute ischemia (age-adjusted hazard ratio, 13 [95% CI, 3–63]).

By May 19, 2020, 9 participants with MRI evidence of acute ischemia and 2 without (age-adjusted hazard ratio, 13 [95% CI, 3–63]) had a new ischemic stroke (Figure 2). Symptoms were equally common at 90 days between participants with and without MRI evidence of acute ischemia, and both groups had similar disability (modified Rankin Scale score of 0–1 acute ischemia: 82%; no acute ischemia: 85% *P*=0.368) and quality of life (EuroQol 0.94 versus 1.00, *P*=0.6038; Table 4).

**Table 4.**
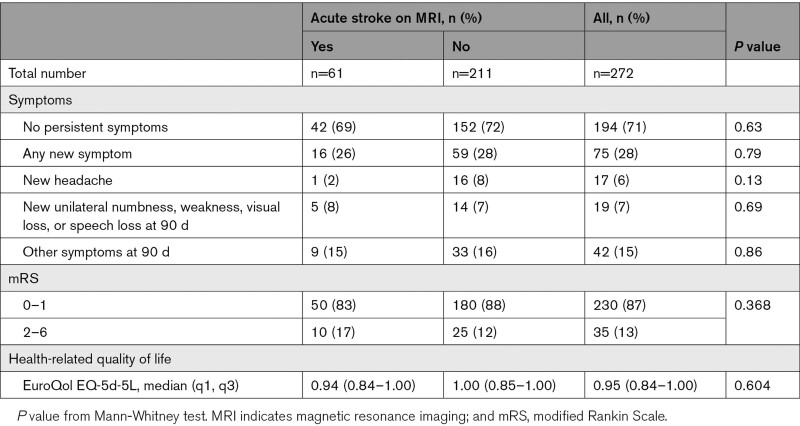
Follow-Up at 90 Days by Letter, Email, or Telephone

## Discussion

In this study, MRI evidence of acute ischemia was seen in 17% (95% CI, 12%–24%) of participants with possible stroke or TIA (where another diagnosis was thought more likely) and 16% (95% CI, 10%–25%) participants with focal symptoms that were not within the National Institute of Neurological Disorders and Stroke definition of TIA. Using the prescription of antiplatelets as a proxy for secondary prevention, we estimated that MRI would have led to a modest increase (5% [95% CI, 3%–8%]) in the number of people who would have been prescribed an antiplatelet, compared with treatment plans without MRI.

The findings from this study are consistent with other literature. In a study of 1028 participants from multiple countries with minor or transient symptoms, 12% of participants with a diagnosis of transient focal neurological events had an MRI DWI finding.^8^ In other smaller studies, DWI-positive lesions were present in 23% (13/56)^10^ of patients with transient neurological attacks and 35% (21/59) patients with neurological symptoms and normal brain computed tomography (CT) presenting to an emergency department.^11^

Clinical diagnosis of transient and minor neurological symptoms relies on a history which may be poorly interpreted by a clinician or remembered with difficulty by patients because of acute or chronic cognitive problems. Therefore, doctors often disagree about whether a patient’s symptoms are due to a TIA when comparing: general practitioners with neurologists,^12^ emergency department doctors with neurologists,^12^ neurologists with neurologists,^13–15^ or even stroke-trained neurologists with stroke-trained neurologists.^16^. Despite this, clinical diagnosis of TIA or stroke is moderately predictive of stroke recurrence in patients presenting to TIA services, even in the absence of brain imaging.^17^ This could improve with a structured proforma for focal symptoms, given that the proportion of people with a DWI lesion was higher in patients with a National Institute of Neurological Disorders and Stroke defined TIA or minor stroke in this study.

The lack of a gold standard diagnosis is a problem in all studies of TIA or minor stroke. People with transient or minor neurological symptoms without a DWI lesion have a higher risk of future stroke than controls. These people may have a normal MRI brain because they are more resilient to brain ischemia, or smaller infarcts that resolve more quickly.^18^

### Strengths

Our study had several strengths. External validity was supported because clinical diagnosis was given by doctors from a range of clinical backgrounds and experience, and participants were recruited from several clinical locations. Internal validity was supported by the small number of participants who did not have an MRI or were lost to follow-up; the recording of a clinical diagnosis before imaging; dual reading of images; and the use of standard proforma to prospectively collect data on symptoms, diagnosis, and demographics.

### Limitations

Our study has some limitations. First, because this was a research study, we could only include participants who consented to take part, which may have led us to recruit a population with a different risk profile to a population-based cohort, despite our efforts to recruit all patients who presented to our service. Compared with all patients arriving at the clinic, these participants were younger (60 versus 69 years) although the distribution of sex and symptoms were similar.^17^ We did not assess further nonconsenting patients. Second, we did not follow every participant up to 24 hours after their onset of symptoms to determine the speed of symptom resolution, and so we could not make a reliable time-defined TIA diagnosis. Third, the sample size was modest and limited by the cost of MRI scanning for research. Fourth, there may have been a Hawthorne effect through observing the diagnostic process, which may have biased preimaging diagnostic probability. Fifth, if we had recruited from a larger number of sites, our findings would have been more generalizable. Sixth, the investigation of stroke cause was clinically driven, and so not all participants were investigated with CT or MR angiography, echocardiography, or prolonged ECG recording. Finally, we were unable to reapproach clinicians to ask whether the results of a scan would have changed their practice. However, the assumption that a positive DWI would change their diagnosis from uncertain to definite is reasonable.

### Implication for Research

The utility of MRI scanning in clinical practice is uncertain, and there have been calls for randomized comparisons of imaging strategies to reduce recurrent stroke risk.^19–21^ However, the sample size for such a study, given the relatively low event rate of stroke in all patients with suspected stroke or TIA, and the modest plausible effect of scanning on recurrent stroke (only acting through additional secondary prevention in the smaller proportion who would not have been diagnosed with a standard strategy), would make any study very large and potentially undeliverable.

### Implications for Practice

Clinical guidelines differ in their recommendations for imaging in patients with suspected TIA or stroke. The National Institute for Health and Care Excellence in the United Kingdom recommend avoiding CT scanning and to consider MRI after specialist assessment to look for alternative pathologies or the territory of ischemia.^21^ The Canadian Stroke Best Practices recommend CT or MRI imaging of brain and major vessels, with the recommended timing of the imaging dependent on clinical symptoms; for patients with focal transient symptoms atypical for ischemia, MRI is recommended within 7 days of symptoms.^22^ The European Stroke Organisation guideline considered there was insufficient evidence to make a recommendation on imaging strategy in TIA or minor stroke.^20^ Lastly, the American Heart Association guidelines recommend CT or MRI of the brain in patients with suspected stroke or TIA, and with a lower quality of evidence, follow-up MRI or CT if the initial imaging is normal.^23^

Health economic studies disagree on whether MR scanning is cost-effective. One study concluded MRI was cost-effective, estimated with an MRI sensitivity of 94% and specificity of 100% (this is an overestimate because only 2/3 of minor stroke have DWI findings), and an estimate of an 80% reduction in risk of recurrent stroke with secondary prevention in the longer term, which may also be an overestimate. In this study, the 30 years costs of an MRI strategy for patients with normal CT were estimated to be $26 304 compared with costs of $27 109 with a CT-only strategy.^24^ However, in a more extensive study, which modeled most of the care pathway, MRI was not cost-effective compared with any other imaging modality (with incremental costs of between £63 and £407).^19^

Countries or regions where access to MRI is not constrained could implement a policy of MRI scanning for all patients with transient or minor neurological symptoms and use the results of MRI to determine subsequent treatment. However, even in the United States only 40% of patients with suspected TIA or stroke receive an MRI within 2 days.^25^. For regions that have limited access to MRI, developing a targeting strategy would be sensible to guide the best use of this resource.

### Conclusions

MRI scanning in all patients presenting with transient or minor neurological symptoms is likely to identify some patients who would otherwise not receive a diagnosis of stroke or TIA because they were otherwise thought to be at low risk. However, whether this strategy is cost-effective, or how MRI scanning could best be targeted, is unclear.

## Article Information

### Sources of Funding

The project was funded by the Chief Scientist’s Office of the Scottish Government (CSO TCS/17/08). Dr Wang was supported by State Scholarship Fund to pursue study in the United Kingdom as a visiting scholar, Fund Number: 201808535094. Dr Whiteley was supported by a Scottish Senior Clinical Fellowship (CAF/17/01). Dr Reed is supported by a National Health Service Research Scotland Career Researcher Clinician award. C. Graham reports grants from the British Heart Foundation. Dr Wardlaw reports grants from Wellcome Trust; grants from Medical Research Council; grants from Fondation Leducq; grants from Dunhill Medical Trust; and grants from Chief Scientist Office. For the purpose of open access, the author has applied a Creative Commons Attribution (CC BY) license to an Author Accepted Manuscript version arising from this submission.

### Disclosures

None.

### Supplemental Material

Table S1

Figure S1

## Supplementary Material


